# Aesthetic preference and gaze-based visual attention in atlas-cover design: Evidence from an eye-tracking study

**DOI:** 10.1371/journal.pone.0359864

**Published:** 2026-10-08

**Authors:** Pizhao Hu, Hao Fang, Yuhui Gui, Nai Yang, Gangqiang Yu, Zixi Huang

**Affiliations:** 1 School of Art and Design, Wuhan Institute of Technology, Wuhan, China; 2 School of Art and Media, Hubei Business College, Wuhan, China; 3 School of Information Engineering, China University of Geosciences (Wuhan), Wuhan, China; Zhejiang Agriculture and Forestry University: Zhejiang A and F University, CHINA

## Abstract

Atlas covers communicate thematic identity and shape first impressions, yet their design remains largely guided by heuristic experience rather than empirical evidence. Although eye-tracking research has examined map use, packaging design, and visual interfaces, little is known about whether atlas-cover variables that enhance perceived attractiveness also align with gaze-based visual-attention indicators. This study addresses this gap by examining how color harmony, graphic style, and layout configuration influence subjective and eye-tracking responses to atlas covers. Forty valid participants viewed eight atlas-cover stimuli generated from a 2×2×2 within-subject factorial design. Each participant completed two counterbalanced viewing cycles, resulting in 16 trials per participant. The two repeated observations for each participant–cover combination were averaged before linear mixed-effects modeling. The results showed that contrast harmony, concrete landscape imagery, and centralized layouts significantly increased perceived attractiveness. The strongest subjective effect was found for graphic style, with landscape images outperforming map symbols (MD=0.66, $p_{Holm}=0.003$), followed by color harmony (MD=0.29, $p_{Holm}<0.001$) and layout configuration (MD=0.14, $p_{Holm}=0.031$). For eye-tracking indicators, centralized layouts produced longer total fixation duration (MD=0.34 s, $p_{Holm}<0.001$) and more fixations (MD=1.13, $p_{Holm}<0.001$). Color harmony showed smaller effects on visual exploration, whereas graphic style mainly affected initial attentional orienting. Mixed-effects association models showed no robust associations between perceived attractiveness and the four gaze-based indicators after Holm–Bonferroni correction (all $p_{Holm}\geq 0.110$). These findings suggest a partial dissociation between aesthetic evaluation and gaze-based visual attention, supporting a dual-layer logic in which layout guides visual organization, while color harmony and graphic style enhance affective appeal and thematic expressiveness.

## Introduction

In the context of information design and cartography, atlases represent not only the systematic integration of spatial data but also a profound fusion of scientific rigor and visual artistry [1]. As the “first point of contact” in the knowledge dissemination chain, the atlas cover often serves as the visual translator of its core content; it functions both as a visual window conveying professional authority and as the gateway guiding readers to establish thematic cognition [2,3]. An effective cover design must balance aesthetic appeal with clear visual organization within a brief first-glance window [4], ensuring that it captures audience attention while supporting rapid identification of the atlas’s thematic positioning. However, for a long time, the creative paradigm for atlas covers has relied excessively on designers’ subjective heuristic experience, resulting in a significant research gap in the cartographic field regarding evidence-based quantitative design principles.

While prior research has accumulated substantial findings on cognitive load related to internal map symbols, colors, and layouts—such as eye-tracking studies revealing fundamental differences in information retrieval patterns between professional cartographers and novices [5,6]—this perspective has rarely extended to the unique medium of atlas covers. In stark contrast, well-developed research in commercial packaging design has confirmed the significant guiding role of variables like color, graphics, and layout in directing consumer attention allocation and decision pathways [7,8]. Furthermore, front-of-package label design for food products has also been shown to substantially influence consumer gaze distribution and selection preferences [9]. This concern has also become increasingly prominent in human–computer interaction (HCI) and user experience (UX) research, where visual artifacts are evaluated not only in terms of stated preference, but also in relation to visual search, perceived complexity, emotional response, and task performance. Recent studies on mobile news applications, online stores, and digital interfaces have shown that visual complexity, interface aesthetics, and the organization of visual elements can alter users’ search behavior, satisfaction, engagement, and decision-making processes [10,11]. These findings suggest that atlas covers, as specialized visual interfaces for geographic information, may follow cognitive principles similar to those observed in packaging, digital interfaces, and other user-facing visual systems.

A core scientific bottleneck remains: we still do not understand how users achieve the cognitive leap from unconscious attraction to meaningful category recognition within hundreds of milliseconds through visual variables such as color, graphics, and layout. Traditional qualitative assessment methods (e.g., interviews or questionnaires) struggle to capture the rapid, continuous, and implicit dynamics of attention competition due to their retrospective limitations [12]. Growing evidence indicates that human visual cognition is not a series of discrete, static snapshots but a dynamic stream unfolding over time [13]. Neurophysiological measurement techniques (e.g., eye tracking) offer the potential to probe this cognitive black box. Eye-tracking metrics can reconstruct the spatial distribution and temporal sequence of gaze behavior, revealing how visual stimuli guide attentional direction and visual exploration [14,15]. This study aims to bridge this gap by proposing an evaluation framework integrating objective eye-tracking data with subjective aesthetic judgments. It systematically analyzes the effects of these variables on the covers of mainstream Chinese atlases over the past 15 years, seeking to provide evidence-based design guidelines for publishing institutions.

## Relevant research and theoretical background

This study’s theoretical framework is rooted in the intersection of cartographic aesthetics and modern cognitive psychology. The evolution of atlas covers not only chronicles advancements in printing technology but also reflects the shifting logic of visual communication [16]. Within the modern GIScience framework, map evaluation criteria have undergone a paradigm shift from “geometric accuracy” to “user cognitive efficiency” [17]. This requires designers to coordinate aesthetic value with clear visual organization while avoiding unnecessary processing demands. A similar shift can be observed in UX design research. User-centered design studies increasingly regard visual interfaces as cognitive environments in which users must identify structure, distinguish visual priority, and form judgments under limited attentional resources [18,19]. In this sense, the atlas cover is not merely an ornamental layer of a cartographic product. It acts as a first-stage information interface through which users infer thematic identity, product credibility, and the expected complexity of the geographic content.

Cognitive Load Theory (CLT) forms the foundational logic of this study. Sweller (1988) noted that the bottleneck of working memory capacity necessitates optimized management of visual stimulus presentation [20]. In cartographic contexts, visual variables not only serve aesthetic purposes but also function as drivers of perceptual salience, governing attention allocation [21]. Empirical studies have demonstrated that the arrangement sequence of layout elements significantly modulates audience attention levels, thereby influencing information retrieval speed [22]. For covers, color and layout may help establish a visual-organizational structure that guides attention during the initial interpretation of geographic information [23]. Recent HCI research further clarifies why layout should be treated as more than a formal aesthetic variable. Eye-tracking evidence from dashboard and mobile interface studies indicates that layout order, element density, and visual complexity can reshape users’ search paths and perceived workload [24,25]. When information is distributed in a coherent and predictable structure, users can allocate attention more efficiently; when visual elements are fragmented or excessive, additional effort is required to locate the target and resolve competing cues. For atlas covers, this suggests that layout may function as a cognitive organizer before users consciously evaluate style or color.

Visual style and color are equally important, but their effects may be more closely related to affective evaluation than to direct cognitive efficiency. Studies of interface aesthetics and emotional experience suggest that visually appealing interfaces can increase pleasure, engagement, and perceived quality, while the relationship between aesthetic pleasure and task efficiency remains contingent on interface structure and task context [26,27]. Research on online store aesthetics also shows that visual presentation can influence the effectiveness of recommendations and the salience of highlighted information, indicating that aesthetic design may shape attention indirectly through perceived relevance and product appeal [28]. In atlas cover design, concrete imagery and color contrast may therefore operate primarily as affective and communicative cues: they enhance the cover’s initial attractiveness and thematic expressiveness, but they may not necessarily reduce the cognitive effort required for geographic information decoding.

The introduction of eye-tracking technology enables the quantification of these implicit processes. Metrics such as total fixation duration (TFD) and fixation count (FC) provide behavioral evidence of visual attention allocation, visual exploration, and processing effort, but they should not be interpreted as direct or exclusive measures of cognitive load without additional physiological or behavioral validation [29]. Recent cutting-edge research has begun exploring the use of multimodal monitoring to dissect neuropsychological mechanisms during geographic information processing [30]. Comparative studies between experts and novices further enrich this theoretical dimension: experts, leveraging well-developed mental schemata, can rapidly extract visual order through more organized scanning paths [31]. This “expert aesthetic” often tends toward higher information tension, while general users exhibit greater tolerance for artistic expression [32]. In broader interface research, eye tracking has become a methodological bridge between subjective UX evaluation and objective behavioral evidence. Systematic reviews of eye-tracking-based UX studies show that gaze indicators are widely used to evaluate usability, attention distribution, and interaction quality [29]. Large-scale datasets covering webpages, desktop interfaces, mobile interfaces, and posters further demonstrate that gaze behavior varies across interface types and is jointly shaped by color, spatial location, and visual composition [33]. These findings justify the use of eye-tracking metrics in this study to capture how users allocate attention to atlas cover elements before forming explicit aesthetic judgments.

The role of user background also deserves attention. Research on interface design for older adults and culturally oriented mobile applications shows that users’ prior knowledge, perceptual habits, and functional expectations can change how they evaluate interface elements such as layout, color, icon style, and interaction mode [34]. Similarly, studies of dynamic GIS interfaces show that information layout can influence search performance in map-related tasks, suggesting that users’ familiarity with spatial information systems may affect how efficiently they interpret interface structures [35]. For atlas covers, professional background may therefore operate not only as a demographic variable, but also as a cognitive schema that shapes sensitivity to visual order, cartographic symbolism, and information tension. This provides a theoretical basis for examining whether professional and non-professional users evaluate the same cover elements differently.

In summary, while cognitive cartography has deep roots in dynamic interface design, systematic empirical evidence remains lacking regarding whether perceived attractiveness is aligned with gaze-based visual-attention indicators in atlas-cover evaluation. The core motivation of this study lies in examining whether the traditional “beauty equals usability” assumption applies to atlas covers as first-stage visual interfaces for geographic information [36].

To address this gap, the present study makes three contributions. First, it extends evidence-based cartographic design from internal map-use tasks to atlas-cover communication by treating the atlas cover as a first-stage visual interface for geographic information products. Second, it integrates perceived attractiveness ratings with eye-tracking indicators within a controlled 2×2×2 factorial design, allowing subjective evaluation and gaze-based visual attention to be examined within the same experimental framework. Third, it directly examines whether aesthetic preference is aligned with gaze-based visual-attention indicators through participant–cover-level mixed-effects association analyses, thereby providing empirical evidence for the partial dissociation between subjective aesthetic evaluation and visual attention.

Based on the above theoretical reasoning, the proposed conceptual model is presented in Fig 1. The model summarizes the direct effects of the three visual design factors on subjective and gaze-based responses, their interaction effects, and the association between perceived attractiveness and gaze-based visual attention. Accordingly, this study proposes the following hypotheses and research question:

**Fig 1:**
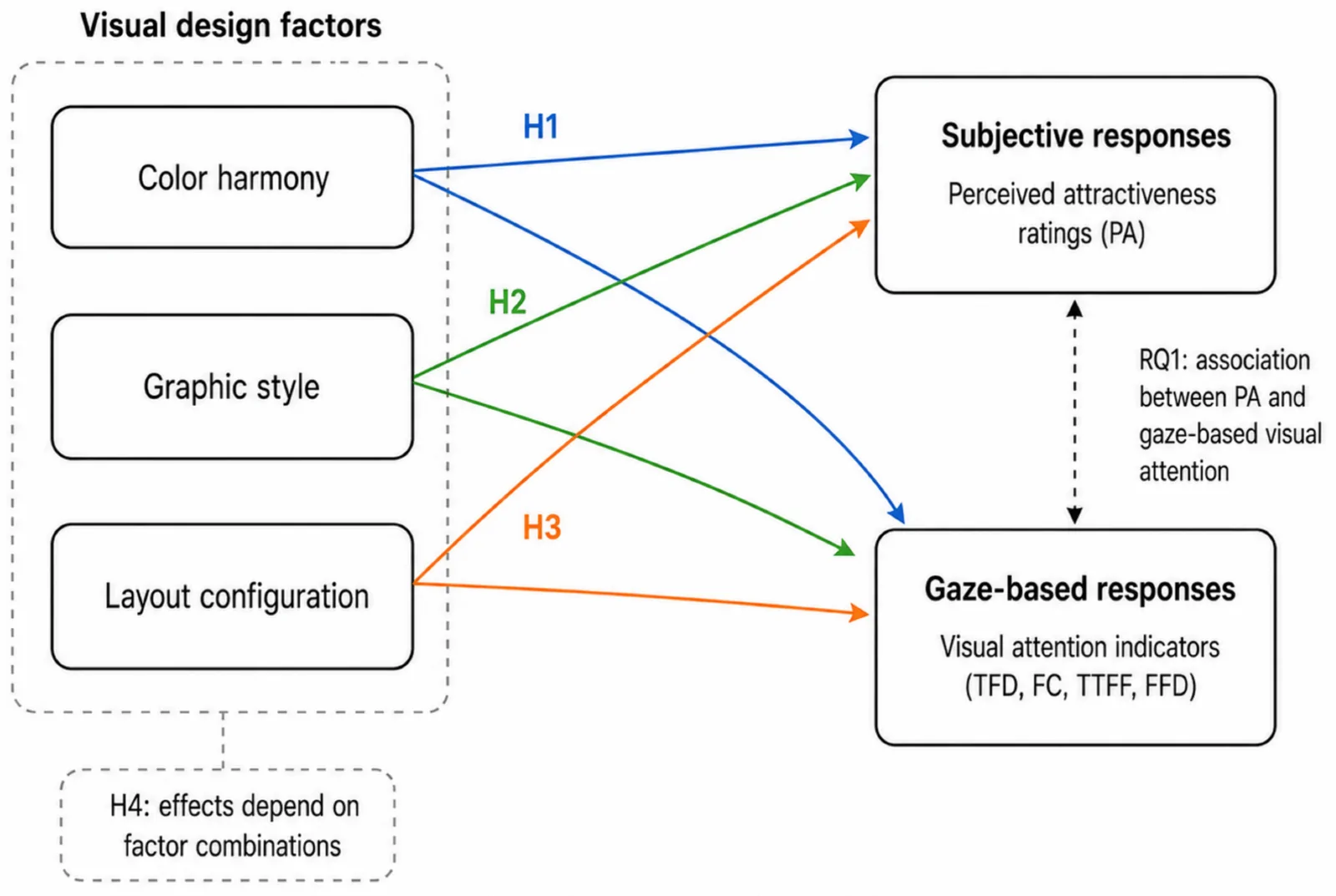
Proposed conceptual model linking visual design factors, subjective responses, gaze-based responses, interaction effects, and the association between perceived attractiveness and visual attention.

H1: Color harmony will influence users’ subjective and gaze-based responses to atlas covers.
H2: Graphic style will influence users’ subjective and gaze-based responses to atlas covers.
H3: Layout configuration will influence users’ subjective and gaze-based responses to atlas covers.
H4: The effects of color harmony, graphic style, and layout configuration will depend on their combinations.
RQ1: How are perceived attractiveness ratings associated with gaze-based indicators of visual attention?

## Materials and methods

### Ethics statement

The experimental protocol of this study was reviewed and approved by the Institutional Review Board (IRB) of Wuhan Institute of Technology (Approval No. WIT-1–20251201). All participants provided written informed consent prior to participation in the experiment. Participation was voluntary, and participants were informed that they could withdraw from the study at any time without penalty. The collected data were used only for academic research purposes and were analyzed anonymously.

### Experimental design and procedure

An indoor eye-tracking experiment was conducted using a VR eye-tracker to record participants’ oculomotor data. During the experiment, participants were placed in a quiet and distraction-free environment. Participants were asked to view atlas covers displayed on a screen within the virtual reality (VR) environment and provide oral ratings of the covers’ attractiveness based on predefined questions. An experimental assistant recorded all responses throughout the procedure.

The experiment adopted a 2×2×2 within-subject factorial design, with color harmony, graphic style, and layout configuration as the three manipulated design factors. The full factorial combination generated eight atlas-cover stimuli, and each participant viewed all eight covers in two counterbalanced viewing rounds. Each participant completed two viewing rounds, with each cover presented once per round, resulting in 16 trials per participant.

In the first round, the covers were presented in the sequence Cover 3, Cover 1, Cover 4, Cover 2, Cover 7, Cover 5, Cover 8, and Cover 6. In the second round, a mirror-balanced counterbalancing sequence was used: Cover 8, Cover 6, Cover 5, Cover 7, Cover 2, Cover 4, Cover 1, and Cover 3. This design was intended to reduce potential order effects and fatigue-related bias.

The 16 questionnaire items were subsequently reorganized according to the actual cover presentation sequence. Questions 5–12 corresponded to the first-round ratings, whereas Questions 13–20 corresponded to the second-round ratings. Thus, each cover was matched with two repeated subjective ratings, one from each round. For each participant, the two ratings corresponding to the same cover were averaged to obtain the final perceived attractiveness score for that cover.

The same participant–cover-level aggregation was applied to the eye-tracking data. Thus, the primary analytical dataset consisted of 320 participant–cover observations, calculated as 40 participants × 8 covers, and repeated viewing cycles were not treated as independent samples. Perceived attractiveness was rated on a seven-point Likert scale ranging from 1, indicating “not attractive at all”, to 7, indicating “extremely attractive”. Finally, the effects of color harmony, graphic style, and layout configuration were examined by integrating subjective attractiveness ratings with objective eye-tracking indicators.

### Participants

A total of 46 participants with normal or corrected-to-normal vision were initially recruited from Wuhan Institute of Technology. The participants ranged in age from 18 to 50 years, with the majority falling within the 20–30 age bracket. To eliminate potential confounding effects of color vision deficiencies on the color harmony variable, all participants underwent a color vision screening using pseudoisochromatic plates. No cases of color blindness or color weakness were identified among the participants retained in the final analysis.

Given that atlases possess both professional and general-interest attributes, the sample comprised both participants with GIS-related backgrounds and lay users from non-professional backgrounds, thereby representing both expert and non-expert perspectives [37]. After excluding participants with incomplete or invalid data or unsuccessful eye-tracking calibration, 40 participants were retained for the final analysis, with 20 participants in the professional group and 20 in the non-professional group.

An *a priori* power analysis was conducted using G*Power 3.1. Sample-size planning was based on the repeated-measures ANOVA procedure for within–between interaction as an approximation for the factorial repeated-measures design. Assuming a medium effect size (*f* = 0.25), α=0.05, power = 0.80, two groups, two repeated measurements, a correlation among repeated measures of 0.50, and a nonsphericity correction of ε=1.00, the required total sample size was 34. Therefore, the final sample of 40 participants exceeded the minimum requirement. The primary inferential analyses were subsequently conducted using linear mixed-effects models to account for repeated observations within participants.

### Apparatus

The eye-tracking component of this experiment utilized the HTC Vive Pro Eye headset (HTC Corp., Taoyuan, Taiwan), which features an integrated eye-tracking module with a sampling frequency of 120 Hz. The ErgoLAB Man-Machine Environment Synchronization and Cloud Platform (Kingfar Technology, Beijing, China) was employed for the experimental design of the VR eye-tracking tasks and the acquisition of oculomotor data.

### Materials

The design of the stimulus materials for this study was based on a quantitative content analysis of 323 atlas covers published by mainstream cartographic publishers over the past 15 years, retrieved from the “China National Publishing and Distribution Public Service Platform” (https://book.cppinfo.cn/). A statistical analysis was performed on the weight distribution of these samples across three categorical dimensions: color harmony, graphic style, and layout configuration. Following a thorough analysis, the most representative levels were selected for experimental manipulation. The levels of color harmony encompassed two categories: analogous vs. contrast harmony. The graphic styles encompassed two categories: concrete landscape vs. map symbols. The layout configurations encompassed two categories: grid vs. centralized layout. The weight analysis for each factor is presented in Table 1.

**Table 1:** Frequency distribution and relative weights of visual design elements in map atlas covers.

Category & Sub-category		Count	Weight
**Color Harmony**	Monochromatic Harmony	58	0.1796
	Analogous Harmony	138	0.4272
	Contrast Harmony	127	0.3932
**Graphics Style**	Concrete Landscape	130	0.4025
	Abstract	40	0.1238
	Map Symbols	153	0.4737
**Layout**	Symmetrical Layout	21	0.0650
	Grid Layout	138	0.4272
	Centralized Layout	141	0.4365
	Diagonal Layout	23	0.0712

Relative weights represent the proportion of each design element relative to the total number of elements within the same category, reflecting their prevalence in the analyzed sample.

The full 2 (color harmony: analogous vs. contrast) × 2 (graphic style: concrete landscape vs. map symbols) × 2 (layout configuration: grid vs. centralized) factorial design resulted in eight unique covers. The assignment of the eight cover stimuli to the three design factors is shown in Table 2.

**Table 2:** Factorial assignment of the eight atlas-cover stimuli.

Cover	Color harmony	Graphic style	Layout configuration
Cover 1	Analogous	Concrete landscape	Grid
Cover 2	Contrast	Concrete landscape	Centralized
Cover 3	Contrast	Concrete landscape	Grid
Cover 4	Analogous	Map symbols	Grid
Cover 5	Analogous	Map symbols	Centralized
Cover 6	Contrast	Map symbols	Grid
Cover 7	Contrast	Map symbols	Centralized
Cover 8	Analogous	Concrete landscape	Centralized

The eight atlas-cover stimuli were generated from a 2×2×2 factorial design, including color harmony, graphic style, and layout configuration as the three manipulated design factors.

To eliminate the interference of confounding variables, all stimulus materials were professionally reconstructed using Adobe Illustrator 2023 (version 27.0, Adobe Inc., San Jose, CA, USA). The reconstructed stimuli were created by the authors for experimental purposes and did not reproduce, trace, or adapt any existing atlas covers, commercial map products, proprietary basemaps, logos, publisher-owned artwork, or third-party copyrighted photographs.

To ensure semantic neutrality, textual information was replaced with non-semantic placeholder characters, such as “XXXX”, thereby mitigating potential semantic confounding arising from text content. In the concrete-landscape condition, the source photographs of landscape and architectural scenes were taken by the authors and subsequently processed into simplified visual materials for the experimental stimuli. No third-party copyrighted photographs were used.

In the map-symbol condition, the Hubei Province outline was redrawn and simplified by the authors with reference to public-domain Natural Earth administrative boundary data and filled with author-created graphic content. The map-like elements were used only as schematic visual-design components for experimental manipulation and were not intended to represent authoritative geographic boundaries or navigation-grade maps. No proprietary basemaps, Google Maps/Earth imagery, OpenStreetMap map tiles, commercial atlas covers, publisher-owned cartographic materials, or copyrighted map images were reproduced or adapted in the stimulus design.

Background luminance was standardized across all stimuli to ensure consistency in low-level visual features, such as contrast and brightness. Seven experts, including scholars in the field of Geographic Information Science (GIScience) and professional designers, were invited to evaluate the sample weight analysis criteria and the stimulus designs using a five-point Likert scale, ranging from 1 = strongly disagree to 5 = strongly agree.

The expert validation results provided robust support for the research design. The overall design received high ratings (*M* = 4.34, *SD* = 0.32), confirming the theoretical soundness and practical utility of the sample weight analysis criteria. The mean effectiveness scores for the eight stimulus designs ranged from 4.54 to 4.75, reflecting a high level of consensus among the experts (refer to Fig 2 for stimulus examples).

**Fig 2:**
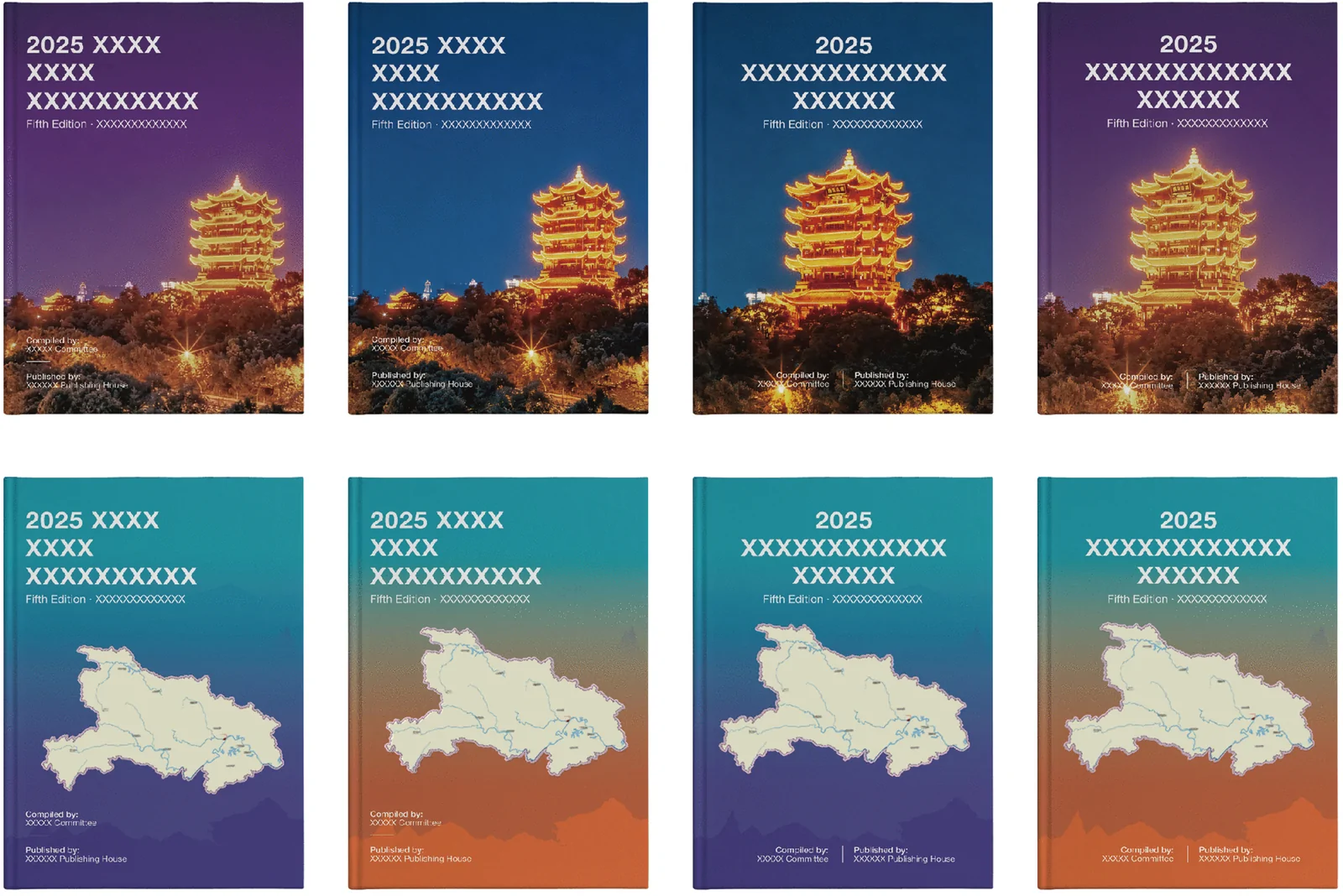
Visual stimuli used in the study. All atlas-cover stimuli were created by the authors for experimental purposes. The landscape source photographs were taken by the authors and processed into simplified visual materials. The Hubei Province outline was redrawn and simplified by the authors with reference to public-domain Natural Earth administrative boundary data. The map-like elements were used only as schematic visual-design components and should not be interpreted as authoritative geographic boundary representations. No proprietary basemaps, Google Maps/Earth imagery, OpenStreetMap map tiles, commercial atlas covers, publisher-owned cartographic materials, or copyrighted map images were reproduced in the stimuli.

### Experimental procedure

As illustrated in Fig 3, the preparation phase began with an introduction to the experimental objectives and procedures, after which participants completed a basic demographic questionnaire. Subsequently, interpupillary distance (IPD) calibration was performed, and participants were allowed a 1–2 minute adaptation period to familiarize themselves with the virtual reality (VR) environment.

**Fig 3:**
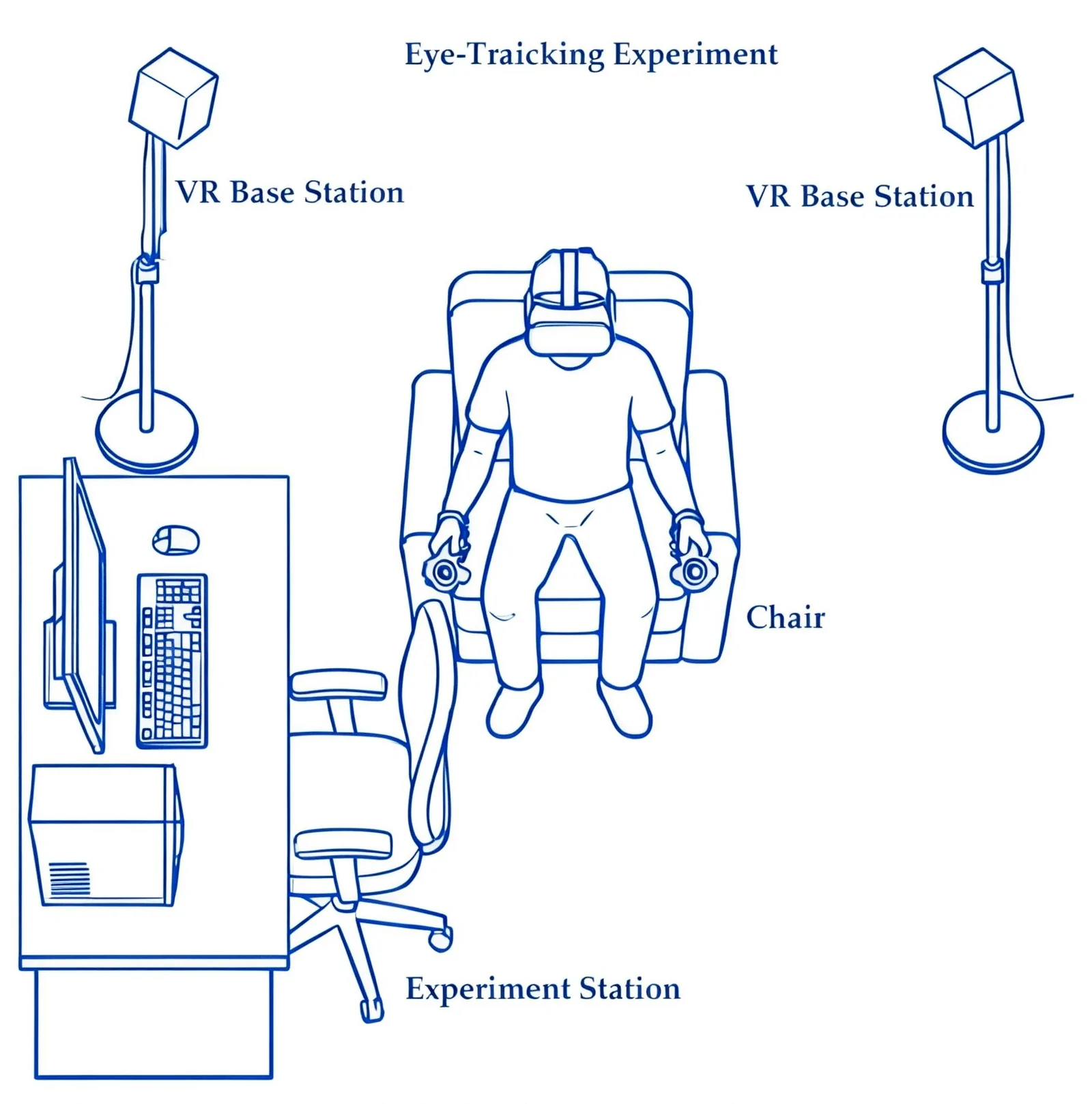
Experimental setup and VR eye-tracking procedure. This figure illustrates the experimental station, VR headset, base stations, and participant viewing environment used for the indoor eye-tracking experiment.

During the formal experimental phase, participants were instructed to view the atlas covers displayed within the VR scene and execute the designated tasks. The stimuli consisted of eight covers presented in fixed counterbalanced orders across two rounds. Each cover was displayed for 5 seconds to simulate the initial perceptual encounter a reader might experience in a physical bookstore or on a digital platform. Following each stimulus presentation, a rating task was triggered. Participants provided oral scores based on the task requirements, which were concurrently recorded by an experimental assistant.

To reduce sequence bias and improve measurement stability, every participant completed two full stimulus-viewing rounds with fixed counterbalanced presentation orders. This mirror-reversal counterbalancing design adopted two standardized stimulus sequences: Round 1 followed the order [1–8], while Round 2 adopted its matched mirror-reversed sequence [1–8]. This arrangement was intended to alleviate systematic order bias and cumulative response fatigue across the experiment. The two counterbalanced rounds were used to improve measurement stability and reduce potential order- and fatigue-related bias, rather than to increase the number of independent observations.

### Analysis metrics and statistical procedures

The analytical framework of this study included two categories of measures: subjective evaluation metrics and eye-tracking metrics. Subjective evaluation was measured using perceived attractiveness (PA), which was rated on a seven-point Likert scale ranging from 1, indicating “not attractive at all”, to 7, indicating “extremely attractive”. Eye-tracking metrics included total fixation duration (TFD, s), fixation count (FC, count), time to first fixation (TTFF, s), and first fixation duration (FFD, s). For AOI-level analyses, the percentage of total fixation duration and the percentage of fixation count were additionally calculated to describe the relative allocation of visual attention across different cover regions. The definitions, units, and analytical roles of these measures are summarized in Table 3.

**Table 3:** Classification and description of subjective and eye-tracking measures.

Category	Measure	Unit	Description / Analytical role
Subjective measure	Perceived attractiveness (PA)	1–7	Subjective rating of atlas-cover attractiveness using a seven-point Likert scale.
Participant variable	Professional background	–	Between-subjects grouping variable, classified as professional vs. non-professional.
Participant variable	Age group	–	Exploratory demographic variable used to examine potential individual differences.
Participant variable	Map-use frequency	–	Exploratory demographic variable reflecting participants’ familiarity with map products.
Eye-tracking metric	Time to first fixation (TTFF)	s	Time elapsed before the first fixation lands within a target cover region or area of interest (AOI).
Eye-tracking metric	First fixation duration (FFD)	s	Duration of the first fixation within a target cover region or AOI.
Eye-tracking metric	Total fixation duration (TFD)	s	Sum of fixation durations within a target cover region or AOI.
Eye-tracking metric	Fixation count (FC)	count	Number of fixations within a target cover region or AOI.
AOI-level metric	TFD share	%	Percentage of total fixation duration allocated to each AOI.
AOI-level metric	FC share	%	Percentage of total fixation count allocated to each AOI.

PA = perceived attractiveness; TTFF = time to first fixation; FFD = first fixation duration; TFD = total fixation duration; FC = fixation count; AOI = area of interest.

For the main eye-tracking analyses, gaze indicators were extracted from the AOI-level statistics exported by ErgoLAB. Specifically, the “Mean” value across the predefined AOIs was used for each participant–cover observation. Therefore, TFD, FC, TTFF, and FFD in the main analyses should be interpreted as AOI-averaged gaze indicators rather than whole-cover totals. This approach ensured that the same extraction rule was applied consistently across all covers and participants. For AOI-level analyses, the same semantic AOI mapping was used for all indicators, and AOIs were not re-ranked separately according to fixation duration, fixation count, or time to first fixation.

Given the factorial repeated-measures structure of the experiment, statistical analyses were primarily conducted using linear mixed-effects models (LMMs) in IBM SPSS 26.0. Each participant viewed multiple atlas covers; therefore, observations from the same participant were not statistically independent. To account for this dependency, participant ID was included as a random intercept in all mixed-effects models. Color harmony, graphic style, layout configuration, and professional background were entered as fixed effects. For the main participant–cover-level analyses, the two repeated observations for the same participant–cover combination were averaged for each dependent variable, yielding 320 participant–cover observations. Viewing cycle was not modeled as a separate fixed factor because the primary research questions concerned the effects of cover design factors rather than repetition effects. This aggregation strategy avoided pseudo-replication and ensured that repeated viewing cycles from the same participant were not treated as independent observations.

For perceived attractiveness, the LMM tested the main effects and interactions of color harmony, graphic style, and layout configuration, as well as the effect of professional background. The same modeling strategy was applied to the eye-tracking indicators, including TFD, FC, TTFF, and FFD. For AOI-level analyses, AOI type was additionally included as a within-subject factor to examine whether attention allocation differed across the main graphic body, main title, secondary information, and auxiliary text areas.

Estimated marginal means were used for planned pairwise comparisons. To control for inflated Type I error due to multiple testing, all planned pairwise comparisons were adjusted using the Holm–Bonferroni procedure. Exploratory demographic analyses involving professional background, age group, and map-use frequency were interpreted cautiously, and multiple-comparison correction was applied where appropriate. To directly examine RQ1 and test the proposed partial dissociation between perceived attractiveness and gaze-based measures, separate linear mixed-effects association models were fitted for TFD, FC, TTFF, and FFD at the participant–cover level. In each model, PA was entered as the dependent variable, the gaze-based indicator was entered as a fixed predictor, and participant ID was specified as a random intercept. Holm–Bonferroni correction was applied across the four association tests.

Model assumptions were examined before interpreting the results. Residual normality was evaluated using Q–Q plots and Shapiro–Wilk tests of model residuals. Homogeneity of variance was assessed by inspecting residuals versus fitted values and, where applicable, by Levene’s tests for between-group comparisons. Because the three focal within-subject factors—color harmony, graphic style, and layout configuration—each had only two levels, the sphericity assumption was automatically satisfied for these comparisons. For AOI analyses involving more than two levels, Mauchly’s test was used to assess sphericity; when sphericity was violated, Greenhouse–Geisser-corrected results were reported.

## Results

This section presents the empirical results of the experiment. Linear mixed-effects models (LMMs) were used for inferential analyses, with participant ID specified as a random intercept to account for repeated observations from the same participants. Unless otherwise specified, all analyses were based on participant–cover-level data after averaging the two viewing rounds for each participant and cover. Descriptive statistics are reported as means and standard deviations, and planned comparisons are summarized using mean differences, 95% confidence intervals, Holm–Bonferroni-adjusted *p* values, and effect sizes where applicable.

### Subjective attractiveness ratings

Perceived attractiveness was first analyzed to examine how the three visual design factors influenced participants’ subjective aesthetic evaluations of atlas covers. Given the repeated-measures structure of the experiment, perceived attractiveness was analyzed using a linear mixed-effects model with participant ID specified as a random intercept. Color harmony, graphic style, layout configuration, and professional background were entered as fixed effects. Planned comparisons were conducted using estimated marginal means, and the resulting *p* values were adjusted using the Holm–Bonferroni procedure.

The LMM results showed that all three visual design factors significantly influenced perceived attractiveness (Table 4 and Fig 4). The largest subjective difference was observed for graphic style, with concrete landscape covers rated higher than map-symbol covers (MD=0.66, $p_{Holm}=0.003$), followed by color harmony, with contrast-harmony covers rated higher than analogous-harmony covers (MD=0.29, $p_{Holm}<0.001$), and layout configuration, with centralized layouts rated slightly but significantly higher than grid layouts (MD=0.14, $p_{Holm}=0.031$).

**Table 4:** Linear mixed-effects model results for perceived attractiveness across design factors.

Design factor	Planned comparison	Mean difference	95% CI	$p_{Holm}$	$\eta_{p}^{2}$
Graphic style	Landscape – Map symbols	0.66	[0.30, 1.02]	0.003	0.26
Color harmony	Contrast – Analogous	0.29	[0.16, 0.42]	<0.001	0.33
Layout configuration	Centralized – Grid	0.14	[0.01, 0.27]	0.031	0.11

Planned comparisons were based on estimated marginal means from linear mixed-effects models. Participant ID was specified as a random intercept. Mean differences were calculated as the first level minus the second level in each comparison. $p_{Holm}$ = Holm–Bonferroni-adjusted *p* value; $\eta_{p}^{2}$ = partial eta-squared. Descriptive statistics were based on participant–cover observations after averaging the two repeated ratings for the same participant–cover combination.

**Fig 4:**
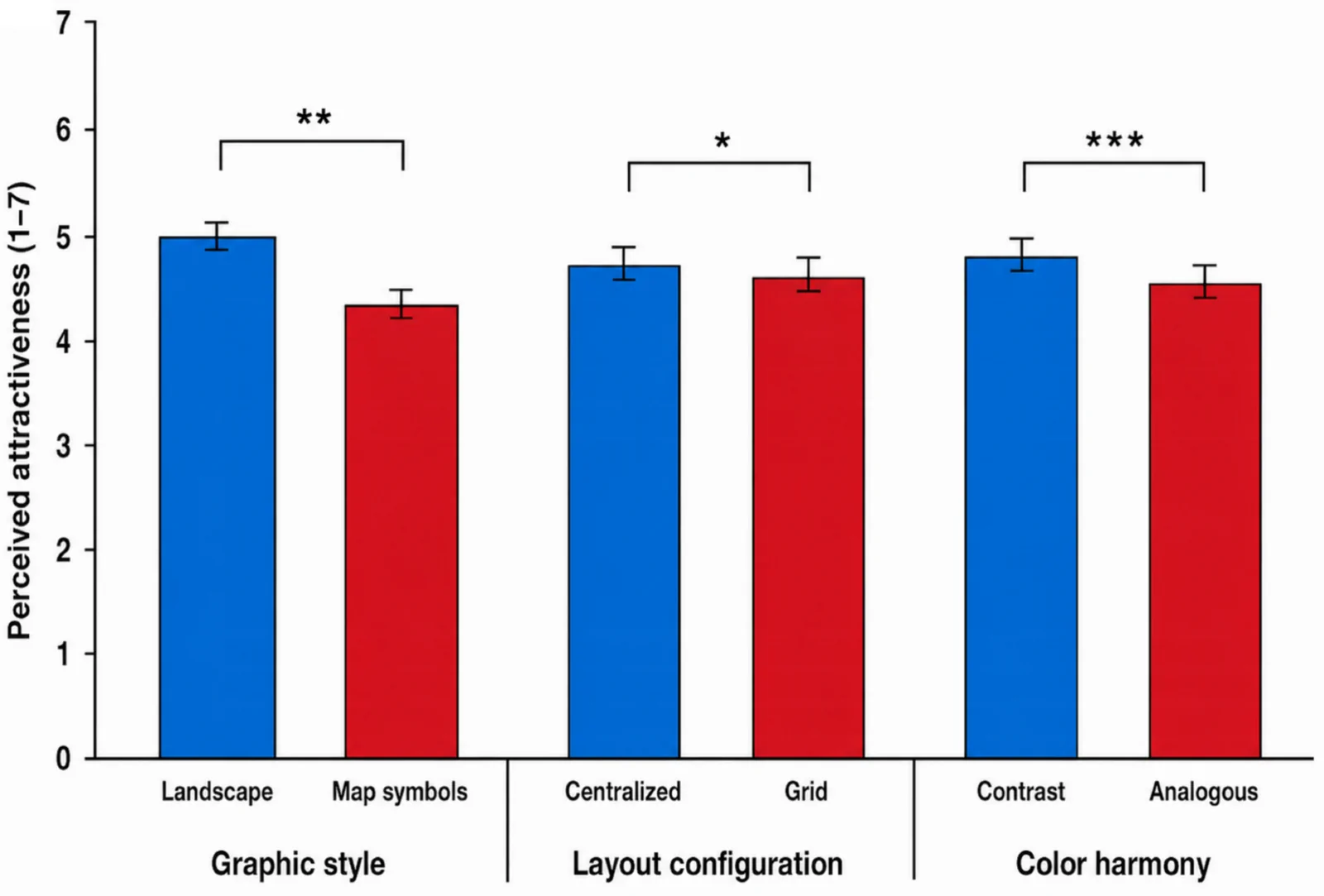
Perceived attractiveness ratings across graphic style, layout configuration, and color harmony. Bars represent mean perceived attractiveness ratings, and error bars represent standard errors. Significance markers are based on Holm–Bonferroni-adjusted planned comparisons from linear mixed-effects models. ^*^p<.05, ^**^p<.01, ^***^p<.001.

Together, these results indicate that all three visual design factors contributed to perceived attractiveness, although their relative magnitudes differed depending on whether raw mean differences or partial eta-squared values were considered. These findings provide support for the perceived-attractiveness component of H1–H3.

### Eye-tracking indicators across design dimensions

Eye-tracking indicators were analyzed to examine whether the three visual design factors influenced visual attention allocation, visual exploration, and initial attentional orienting. Total fixation duration (TFD), fixation count (FC), time to first fixation (TTFF), and first fixation duration (FFD) were treated as gaze-based outcome variables, with color harmony, graphic style, and layout configuration specified as the primary design predictors.

Because each stimulus was displayed for 5 s, the eye-tracking indicators were directly comparable across covers. Fig 5 provides a descriptive visualization of fixation-density distributions across the eight atlas-cover stimuli. The heatmaps show that visual attention was generally concentrated around the central graphic body and title-related regions, although the degree and spatial pattern of fixation concentration varied across design configurations. These visual patterns provide an initial descriptive overview and were further examined using quantitative eye-tracking indicators in the subsequent analyses.

**Fig 5:**
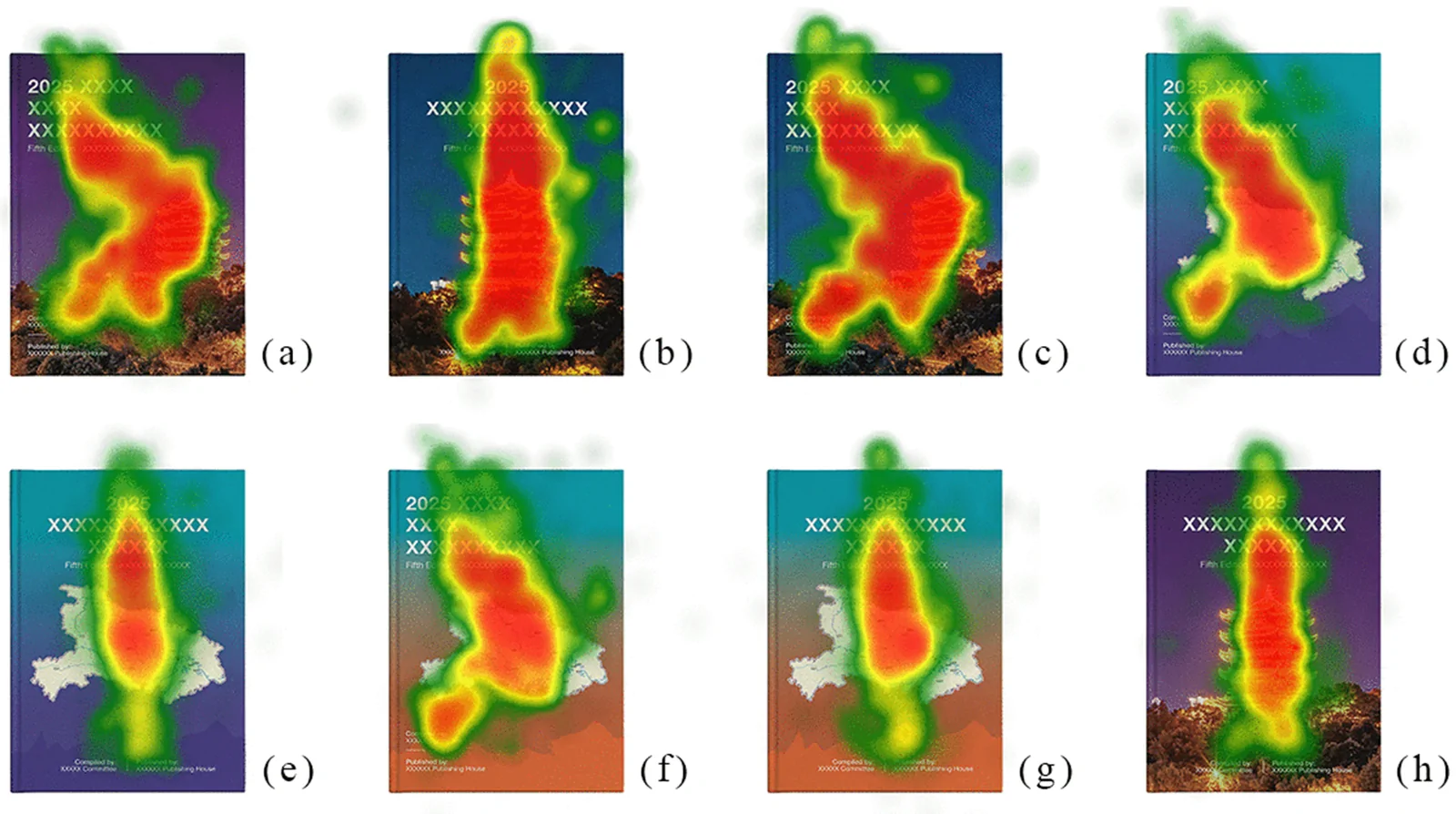
Eye-tracking heatmaps for the eight atlas-cover stimuli. Panels (a)–(h) correspond to Covers 1–8, respectively. Warmer colors indicate higher fixation density during the 5 s viewing period. The stimulus thumbnails were created by the authors for experimental purposes. Landscape photographs were taken and processed by the authors, and the Hubei Province outline was redrawn and simplified with reference to public-domain Natural Earth administrative boundary data. The map-like elements are schematic and do not reproduce proprietary basemaps, Google Maps/Earth imagery, OpenStreetMap map tiles, commercial atlas covers, or copyrighted map images.

Descriptive statistics showed that layout configuration produced the clearest differences in visual attention indicators. Centralized layouts elicited longer total fixation duration and more fixations than grid layouts. Color harmony also showed smaller differences, with contrast-harmony covers producing slightly longer fixation duration and more fixations than analogous-harmony covers. In contrast, graphic style showed limited differences in TFD and FC but was associated with a difference in TTFF, with map-symbol covers showing shorter TTFF than landscape covers (Table 5).

**Table 5:** Descriptive statistics of AOI-averaged eye-tracking indicators across design factors.

Design factor	Level	N	TFD, M ± SD	FC, M ± SD	TTFF, M ± SD	FFD, M ± SD
Layout configuration	Centralized	160	2.34 ± 0.49	8.78 ± 1.99	0.94 ± 0.46	0.26 ± 0.13
Layout configuration	Grid	160	1.99 ± 0.51	7.65 ± 1.91	1.05 ± 0.54	0.24 ± 0.11
Graphic style	Landscape	160	2.18 ± 0.56	8.16 ± 2.21	1.09 ± 0.51	0.26 ± 0.13
Graphic style	Map symbols	160	2.15 ± 0.49	8.27 ± 1.82	0.90 ± 0.48	0.24 ± 0.11
Color harmony	Analogous	160	2.13 ± 0.51	7.97 ± 1.93	0.97 ± 0.53	0.25 ± 0.12
Color harmony	Contrast	160	2.20 ± 0.55	8.46 ± 2.10	1.01 ± 0.48	0.25 ± 0.11

TFD = total fixation duration; FC = fixation count; TTFF = time to first fixation; FFD = first fixation duration. TFD, TTFF, and FFD are measured in seconds, whereas FC is measured as fixation count. Descriptive statistics are based on participant–cover observations after averaging repeated observations for the same participant–cover combination.

The LMM results confirmed that layout configuration exerted the most consistent influence on visual attention indicators. The layout effect was also numerically larger than the color effect: centralized layouts increased TFD by 0.34 s and FC by 1.13 fixations relative to grid layouts, whereas contrast harmony increased TFD by 0.07 s and FC by 0.49 fixations relative to analogous harmony (Table 6). Centralized layouts produced significantly longer TFD than grid layouts and also elicited significantly higher FC, indicating more sustained and extensive visual exploration. Color harmony also had significant but smaller effects on TFD and FC, with contrast-harmony covers receiving slightly more visual attention than analogous-harmony covers. Graphic style did not significantly alter TFD or FC but influenced TTFF, with map-symbol covers producing a 0.19 s shorter TTFF than landscape covers. No robust main effects were observed for FFD. Significant planned comparisons are reported in Table 6.

**Table 6:** Linear mixed-effects model results for significant eye-tracking indicators across design factors.

DV	Design factor	Planned comparison	MD	95% CI	$p_{Holm}$	$\eta_{p}^{2}$
TFD	Layout configuration	Centralized – Grid	0.34	[0.27, 0.41]	<0.001	0.69
FC	Layout configuration	Centralized – Grid	1.13	[0.74, 1.52]	<0.001	0.47
TFD	Color harmony	Contrast – Analogous	0.07	[0.02, 0.14]	0.031	0.14
FC	Color harmony	Contrast – Analogous	0.49	[0.23, 0.75]	0.003	0.27
TTFF	Graphic style	Map symbols – Landscape	−0.19	[−0.31, −0.07]	0.006	0.21

DV = dependent variable; MD = mean difference. Planned comparisons were based on estimated marginal means from linear mixed-effects models. Participant ID was specified as a random intercept. Mean differences are calculated as the first level minus the second level in each comparison. $p_{Holm}$ = Holm–Bonferroni-adjusted *p* value; $\eta_{p}^{2}$ = partial eta-squared. TFD and TTFF are measured in seconds, and FC is measured as fixation count. Only statistically significant planned comparisons are shown.

Overall, these results indicate that layout configuration was the strongest and most stable predictor of eye-tracking responses, particularly for TFD and FC. Color harmony produced smaller but significant effects on visual exploration, whereas graphic style mainly influenced the speed of initial visual entry rather than the overall amount of attention allocated to the cover. These findings provide partial support for the gaze-based components of H1–H3. Layout configuration showed the strongest and most consistent gaze-based effects, color harmony showed smaller effects on visual exploration, whereas graphic style selectively influenced initial attentional orienting rather than overall fixation duration or fixation count.

### Interaction effects among design factors

Interaction effects among the three visual design factors were further examined to determine whether the effect of one design variable depended on the configuration of another. The interaction terms among color harmony, graphic style, and layout configuration were tested using the same linear mixed-effects modeling framework described above. Interaction effects with nominal or corrected statistical significance are reported in Table 7, with Holm–Bonferroni-adjusted *p* values provided where applicable.

**Table 7:** Significant interaction effects among design factors based on linear mixed-effects models.

Dependent variable	Interaction term	*F*(1,39)	*p*	$p_{Holm}$	$\eta_{p}^{2}$	Interpretation
PA	Layout configuration × Color harmony	5.52	0.024	0.072	0.12	The effect of color harmony on attractiveness varied by layout configuration.
TFD	Graphic style × Layout configuration	32.56	<0.001	<0.001	0.46	The layout effect on fixation duration differed by graphic style.
TFD	Graphic style × Color harmony	6.83	0.013	0.026	0.15	The color effect on fixation duration differed by graphic style.
FC	Graphic style × Layout configuration	26.52	<0.001	<0.001	0.41	The layout effect on fixation count differed by graphic style.
FC	Graphic style × Color harmony	18.78	<0.001	<0.001	0.33	The color effect on fixation count differed by graphic style.

PA = perceived attractiveness; TFD = total fixation duration; FC = fixation count; $\eta_{p}^{2}$ = partial eta-squared. Interaction effects were tested using linear mixed-effects models with participant ID specified as a random intercept. The PA interaction was nominally significant but did not survive Holm–Bonferroni correction; therefore, it was interpreted cautiously. Other listed eye-tracking interactions remained significant after correction.

For perceived attractiveness, a nominal interaction was observed between layout configuration and color harmony. This result suggests that the effect of color harmony on subjective attractiveness may vary across layout configurations. However, this interaction did not survive Holm–Bonferroni correction and should therefore be interpreted cautiously.

For eye-tracking indicators, significant interaction effects were mainly observed for TFD and FC. Specifically, the interaction between graphic style and layout configuration was significant for both TFD and FC, indicating that the effect of layout configuration on visual exploration differed between landscape covers and map-symbol covers. In addition, the interaction between graphic style and color harmony was significant for both TFD and FC, suggesting that the influence of color harmony on fixation duration and fixation count was contingent on the graphic style of the cover. No robust interaction effects were observed for FFD.

Taken together, these interaction results indicate that the effects of visual design variables were not entirely additive. Layout configuration remained the most stable predictor of visual attention, but its influence was partly shaped by graphic style. Similarly, color harmony affected subjective attractiveness and visual exploration, although the interaction involving subjective attractiveness should be interpreted cautiously after correction. These findings provide partial support for H4. Robust interaction effects were observed for TFD and FC, whereas the interaction involving perceived attractiveness was only nominal and should be interpreted cautiously after correction.

### AOI-level attention distribution

To further examine how visual attention was distributed across different regions of the atlas covers, AOI-level analyses were conducted for four predefined semantic areas of interest: the main graphic body (I1), the main title (T1), secondary information (I2), and auxiliary text areas (T2). The AOI labels were assigned before statistical analysis according to the semantic function of each cover region. The same AOI mapping was used for all AOI-level indicators, including TFD, FC, TFD share, FC share, and TTFF.

Descriptive statistics indicated a highly uneven distribution of visual attention across AOIs (Table 8). The main graphic body (I1) attracted the largest proportion of visual attention, accounting for more than half of both total fixation duration and fixation count. The main title area (T1) received the second-highest level of attention, followed by secondary information (I2). Auxiliary text areas (T2) attracted very little attention during the brief cover-viewing task. In terms of temporal sequence, I1 was fixated earliest, followed by T1, whereas I2 and T2 were accessed substantially later.

**Table 8:** AOI-level distribution of visual attention.

AOI	Region	TFD, M ± SD	TFD share, M ± SD (%)	FC, M ± SD	FC share, M ± SD (%)	TTFF, M ± SD
I1	Main graphic body	4.02 ± 1.80	54.38 ± 19.99	14.54 ± 5.06	53.21 ± 18.88	0.40 ± 0.59
T1	Main title	1.46 ± 1.01	20.95 ± 14.43	6.06 ± 4.19	20.98 ± 13.39	1.10 ± 1.03
I2	Secondary information	0.97 ± 0.84	13.33 ± 10.96	3.79 ± 3.06	13.49 ± 10.45	1.89 ± 1.20
T2	Auxiliary text areas	0.13 ± 0.33	1.76 ± 4.36	0.53 ± 1.20	1.75 ± 3.95	2.46 ± 1.03

AOI = area of interest; TFD = total fixation duration; FC = fixation count; TTFF = time to first fixation. TFD and TTFF are measured in seconds, whereas FC is measured as fixation count. TFD share and FC share indicate the percentage of total fixation duration and fixation count allocated to each AOI. AOIs were semantically mapped across covers into four comparable regions, and the same AOI mapping was used for all AOI-level indicators.

Linear mixed-effects models with AOI type as a within-subject factor confirmed significant differences in attention allocation across the four AOIs. Significant main effects of AOI type were observed for TFD, *F*(3,117) = 135.08, *p* < 0.001; FC, *F*(3,117) = 197.49, *p* < 0.001; TFD share, *F*(3,117) = 173.90, *p* < 0.001; and FC share, *F*(3,117) = 184.17, *p* < 0.001. Pairwise comparisons with Holm–Bonferroni correction showed that I1 received significantly greater attention than T1, I2, and T2, whereas T2 received significantly less attention than all other AOIs. These results indicate that participants’ visual attention was primarily anchored by the main graphic body, with the main title functioning as a secondary information entry point.

### Direct association between subjective attractiveness and eye-tracking indicators

To examine whether perceived attractiveness was associated with gaze-based indicators while accounting for the repeated-measures structure, mixed-effects association models were fitted. In each model, perceived attractiveness was entered as the dependent variable, one gaze-based indicator was entered as the fixed predictor, and participant ID was specified as a random intercept. Separate models were estimated for TFD, FC, TTFF, and FFD. Holm–Bonferroni correction was applied across the four association tests.

The results showed that perceived attractiveness was not robustly associated with the main gaze-based indicators after accounting for the repeated-measures structure. Although FC showed a nominal positive association with perceived attractiveness before correction, this association did not remain statistically significant after Holm–Bonferroni correction. TFD, TTFF, and FFD also showed no significant associations with perceived attractiveness. These findings indicate that higher subjective attractiveness ratings did not necessarily correspond to longer fixation duration, more fixations, shorter time to first fixation, or longer first fixation duration (Table 9).

**Table 9:** Mixed-effects associations between perceived attractiveness and eye-tracking indicators.

Predictor	N	b	SE	t	*p*	$p_{Holm}$
TFD	320	0.183	0.112	1.639	0.101	0.304
FC	320	0.060	0.027	2.203	0.028	0.110
TTFF	320	0.100	0.101	0.994	0.320	0.640
FFD	320	0.261	0.428	0.609	0.542	0.640

PA = perceived attractiveness; TFD = total fixation duration; FC = fixation count; TTFF = time to first fixation; FFD = first fixation duration. Separate mixed-effects association models were fitted for each gaze-based predictor. In each model, PA was entered as the dependent variable, the gaze-based indicator was entered as a fixed predictor, and participant ID was specified as a random intercept. $p_{Holm}$ = Holm–Bonferroni-adjusted *p* value.

Taken together, these results suggest that subjective attractiveness and eye-tracking indicators captured related but not equivalent aspects of atlas-cover evaluation. While visual design factors influenced both subjective ratings and eye-tracking responses, the non-significant adjusted associations between PA and eye-tracking metrics indicate that aesthetic preference and visual-attention allocation were only partially aligned. These findings directly address RQ1 and provide statistical evidence for a partial dissociation between subjective aesthetic evaluation and gaze-based visual-attention measures.

### Exploratory demographic analyses

To complement the main analyses, additional exploratory models were conducted to examine whether participant-level characteristics were associated with perceived attractiveness. These analyses focused on professional background, age group, and map-use frequency. Because these variables were not part of the primary experimental design, they were treated as exploratory predictors rather than confirmatory factors.

Each demographic variable was entered as a between-subjects fixed effect in separate linear mixed-effects models, with participant ID specified as a random intercept. Perceived attractiveness was used as the dependent variable. The exploratory fixed-effect results are summarized in Table 10.

**Table 10:** Exploratory fixed effects of demographic variables on perceived attractiveness.

Predictor	Fixed-effect contrast	Estimated mean difference	*F*	df	*p*	Effect size
Professional background	Professional – Non-professional	0.23	3.51	1, 38	0.069	$\eta^{2}=0.085$
Age group	Younger – Older	−0.13	0.94	1, 38	0.339	$\eta^{2}=0.024$
Map-use frequency	High – Low	0.13	1.00	1, 38	0.323	$\eta^{2}=0.026$

Demographic variables were analyzed as exploratory between-subjects fixed effects in linear mixed-effects models, with participant ID specified as a random intercept. Estimated mean differences are calculated as the first level minus the second level in each contrast. Age group was dichotomized as younger versus older participants, and map-use frequency was dichotomized as high versus low frequency for exploratory comparison. These analyses were exploratory and should be interpreted cautiously.

Professional background showed a small tendency toward higher perceived attractiveness ratings among participants with professional experience, but this effect did not reach statistical significance. Similarly, age group and map-use frequency did not show robust effects on perceived attractiveness. These results suggest that demographic characteristics may contribute to individual variation in aesthetic evaluation, but their effects were weaker and less stable than the main experimental design factors.

Importantly, these exploratory analyses do not provide evidence for a confirmed moderating role of professional background, age group, or map-use frequency. A moderation effect would require stable interactions between these participant-level variables and the focal design factors. Therefore, the demographic findings are interpreted as preliminary evidence of individual differences rather than as primary explanatory results.

## Discussion

This study examined how color harmony, graphic style, and layout configuration shape atlas-cover evaluation by combining perceived attractiveness ratings with eye-tracking indicators. The findings show that atlas-cover design cannot be understood solely as an aesthetic problem or solely as an information-decoding problem. Instead, subjective preference and gaze-based visual attention reflected related but non-equivalent dimensions of cover evaluation. Specifically, all three design factors influenced perceived attractiveness, whereas eye-tracking indicators showed a more selective pattern: layout configuration produced the strongest and most consistent effects, color harmony produced smaller but significant effects on visual exploration, and graphic style mainly influenced the speed of initial visual entry. These findings provide empirical support for a dual-layer interpretation of atlas-cover design, in which layout organizes attention and information hierarchy, while color and graphic style contribute primarily to affective appeal and thematic expression.

### Partial dissociation between aesthetic preference and visual attention

A central finding of this study is that perceived attractiveness was only weakly associated with eye-tracking indicators. None of the adjusted associations between perceived attractiveness and TFD, FC, TTFF, or FFD remained significant after Holm–Bonferroni correction. This result addresses RQ1 and suggests that covers rated as more attractive were not necessarily those that received longer fixation duration, more fixations, shorter time to first fixation, or greater relative attention allocation.

This finding refines the classic assumption that “what is beautiful is usable”. Tractinsky et al. proposed that users often infer usability from visual beauty, especially during rapid interface evaluation [38]. However, the present study shows that this relationship is not straightforward in atlas-cover design. A cover can be aesthetically preferred without necessarily producing more sustained, more extensive, or more rapidly oriented gaze responses. This difference may arise because atlas covers are not conventional task interfaces: they function simultaneously as symbolic, commercial, and cartographic objects. In such a context, subjective attractiveness may capture affective impression and thematic appeal, while eye-tracking indicators capture the distribution and sequence of visual inspection.

The present result is partly consistent with Reppa and McDougall’s finding that aesthetic appeal can influence visual search performance [4]. Their study suggests that beauty may affect visual search under certain task conditions. In contrast, our results show that aesthetic preference did not translate into a general improvement or reduction in gaze-based indicators across atlas covers. This difference may be due to the task structure. In visual search tasks, users often search for specific targets, whereas in the present study participants viewed covers briefly and evaluated attractiveness. Under this condition, gaze behavior may reflect initial exploration rather than goal-directed search efficiency.

The result is also consistent with broader evidence that aesthetic judgments and cognitive judgments may rely on partly different processing resources. Bara et al. showed that aesthetic and non-aesthetic judgments do not necessarily place identical demands on working memory resources [39]. This supports our interpretation that subjective attractiveness and gaze-based visual attention should not be treated as interchangeable measures. In the present study, perceived attractiveness captured the evaluative response to the cover as a whole, whereas eye-tracking indicators captured how visual attention was distributed during brief exposure.

From the perspective of cognitive load theory, this partial dissociation is theoretically meaningful. Sweller’s cognitive load theory emphasizes that working memory limitations make information organization critical during visual processing [20]. Bunch and Lloyd extended this logic to geographic information and argued that geographic displays can impose cognitive load through their structure and information complexity [40]. In the present study, visually attractive features such as contrast harmony or landscape imagery may have enhanced affective appeal, but they did not uniformly reduce or increase visual-processing demands. This suggests that beauty and cognitive organization operate through related but distinct mechanisms in atlas-cover perception.

### Layout configuration as the strongest visual-organizational scaffold

The most stable result across subjective and eye-tracking measures concerns layout configuration. Centralized layouts received higher perceived attractiveness ratings than grid layouts and produced longer total fixation duration and more fixations. This result indicates that layout configuration influenced not only how participants evaluated the cover, but also how they visually explored it.

This finding is consistent with visual search theory. Wolfe and Horowitz argued that visual attention is guided by multiple factors, including spatial layout, salience, and scene structure [23]. In the present study, centralized layouts may have provided a clearer spatial anchor, making it easier for participants to locate the main graphic body and title-related information. Rather than scattering attention across multiple competing regions, the centralized structure likely created a stable focal hierarchy.

The result also aligns with packaging and interface studies showing that the position and organization of design elements influence visual attention. Barbosa et al. found that the placement of design elements on packaging affected consumers’ attention levels [22]. Similarly, Zhang et al. showed through eye-tracking evidence that dashboard layout order can influence interface complexity and users’ search behavior [24]. These findings support the present interpretation that layout is not merely a formal aesthetic attribute; it actively structures how users allocate attention.

In GIS-related interface design, Shao et al. found that dynamic information layout affects users’ search performance in GIS interfaces [35]. Although their study focused on interactive GIS information rather than static atlas covers, both findings point to the same principle: spatial organization determines how efficiently users locate and interpret visually distributed information. For atlas covers, this means that layout configuration operates as a first-level visual-organizational scaffold before users engage in more detailed aesthetic or semantic interpretation.

At the same time, layout should not be described as the sole factor influencing atlas-cover evaluation. The present study shows that layout had the strongest and most consistent effects, but color harmony and graphic style also contributed to specific aspects of subjective and gaze-based responses. Therefore, the more accurate conclusion is that layout functions as the primary organizer of visual attention, while color and style shape more selective affective and perceptual responses.

### Selective effects of color harmony and graphic style

Color harmony significantly influenced perceived attractiveness, with contrast harmony receiving higher ratings than analogous harmony. It also produced small but significant effects on TFD and FC. This result suggests that color contrast increased visual exploration to some extent, possibly because it enhanced perceptual salience and visual tension.

This finding is consistent with interface and visual design research showing that color can influence perception and user experience. Ma et al. found that interface color quantity and similarity can affect visual perception, using EEG evidence to show that color organization changes perceptual processing [26]. In the present study, contrast harmony may have enhanced perceptual distinctiveness, making the cover visually more engaging and increasing fixation duration and fixation count. However, the effect of color was smaller than that of layout, suggesting that color is better understood as a salience-enhancing cue rather than the primary organizer of visual attention.

Sun and Jiang reported that eye movements are related to users’ emotional experience in interface design [27]. This supports the interpretation that color harmony may influence both affective evaluation and gaze behavior. However, our study further shows that such effects are selective. Color contrast increased subjective attractiveness and some visual exploration indicators, but it did not uniformly affect all eye-tracking metrics. This nuance is important because it prevents an overgeneralized claim that color either “does” or “does not” influence gaze behavior.

Graphic style showed a different pattern. Concrete landscape covers received higher perceived attractiveness ratings than map-symbol covers, suggesting that representational imagery may produce stronger immediate affective appeal. However, graphic style did not significantly alter TFD or FC. Instead, map-symbol covers showed shorter TTFF than landscape covers, indicating faster initial visual entry.

This finding is consistent with cartographic cognition research showing that map symbols and map-related visual structures can shape recognition and attention. Keskin et al. found that expertise influences visual attention and recognition in map-reading and memorability tasks [31]. In the present study, map-symbol covers may have offered more schematic and spatially recognizable cues, allowing viewers to enter the visual structure more quickly. At the same time, concrete landscapes may have been more aesthetically appealing because they provided richer imagery and stronger thematic associations.

Therefore, color harmony and graphic style should not be characterized as irrelevant to visual attention. Their effects were selective rather than absent. Color harmony influenced both perceived attractiveness and visual exploration, while graphic style influenced perceived attractiveness and the speed of initial visual entry. These results suggest that atlas-cover design requires a balance between affective communication and efficient visual orientation.

### Configuration-dependent effects among design variables

The interaction results suggest that visual design variables did not operate in a purely additive manner. Robust interaction effects were observed in eye-tracking indicators, especially for TFD and FC. The interaction between graphic style and layout configuration indicates that the effect of layout on visual exploration differed depending on whether the cover used concrete landscape imagery or map symbols. The interaction between graphic style and color harmony further indicates that the effect of color on fixation duration and fixation count depended on graphic style.

This result is consistent with the idea that visual attention is shaped by combinations of cues rather than isolated features. Wolfe and Horowitz emphasized that visual search is guided by multiple interacting factors rather than a single visual attribute [23]. In the present study, layout, color, and graphic style jointly shaped the visual field of the cover. A centralized layout may organize attention differently when paired with concrete imagery than when paired with map symbols, and color contrast may have different effects depending on the visual form it supports.

The finding also resonates with interface design research showing that visual complexity and element organization jointly influence search behavior. Guo et al. found that visual complexity in mobile news applications affects users’ search behavior and satisfaction [10]. Although their study focused on digital interfaces, it provides a useful parallel: users respond to the overall configuration of visual elements, not to isolated elements alone. In atlas covers, the same principle applies because cover perception depends on how layout, imagery, and color are combined into a coherent visual structure.

For perceived attractiveness, the interaction between layout configuration and color harmony was nominally significant but did not survive Holm–Bonferroni correction. Therefore, this effect should be interpreted cautiously. It may indicate a possible tendency for color effects to vary across layouts, but the present data do not provide sufficiently robust evidence for a strong subjective interaction. This distinction is important because it keeps the interpretation aligned with the corrected statistical results.

Overall, the interaction findings provide partial support for H4. They show that design variables are not completely independent, particularly in gaze-based measures. For practical atlas-cover design, this means that layout, color, and graphic style should be coordinated as a system rather than optimized separately.

### Exploratory individual differences

Professional background, age group, and map-use frequency were examined as exploratory participant-level variables. The results did not provide evidence for robust demographic moderation. Professional background showed only a non-significant tendency toward higher perceived attractiveness ratings among participants with professional experience, while age group and map-use frequency did not show stable effects.

This result differs from stronger claims in previous map-use research that expert and novice users may process maps differently. Ooms et al. showed that expert and novice users can differ in how they interpret maps through eye movement patterns [5]. Keskin et al. also found expertise-related differences in visual attention and recognition during map-reading and memorability tasks [31]. Compared with these studies, the present study found weaker expertise-related effects. One likely reason is that the present task involved brief atlas-cover evaluation rather than complex map reading or spatial reasoning. Expertise may become more influential when users need to decode map content, compare spatial relations, or solve geographic problems.

Keskin et al. further showed that EEG and eye-tracking measures can reveal cognitive-load differences between expert and novice map users [30]. In the present study, however, professional background did not produce a robust moderating effect on perceived attractiveness. This difference may reflect the level of task demand. Atlas-cover viewing may rely more on general visual preference and first impressions, whereas expert advantages may emerge more clearly in tasks requiring domain-specific interpretation.

Therefore, professional background should be interpreted as a potential source of individual differences rather than as a confirmed moderator. This exploratory pattern remains theoretically meaningful because GIS-related or design-related experience may still shape sensitivity to visual order, map symbolism, and information hierarchy. However, stronger conclusions require larger samples, more balanced demographic groups, and more fine-grained measures of expertise.

### Implications for cartographic design

The findings support a dual-layer design logic for atlas covers. The first layer is visual organization, primarily supported by layout configuration. Designers should use layout to establish a clear visual hierarchy, guide attention to the main graphic body and title, and reduce ambiguity during initial viewing. This implication is consistent with geovisualization research emphasizing that the effectiveness of geographic information displays depends on perceptual salience, visual organization, and usability [41].

The second layer is affective and communicative expression, primarily supported by color harmony and graphic style. Contrast harmony can increase visual salience and perceived attractiveness, while concrete landscape imagery can strengthen thematic expressiveness. However, these elements should be coordinated with layout rather than used as isolated decorative features. This point is consistent with mobile map design research, which emphasizes that cartographic interfaces should adapt design choices to context, user needs, and map-use conditions [42].

For evidence-based atlas-cover design, the practical implication is that subjective preference alone is insufficient. Eye-tracking-based UX research has shown that gaze indicators can provide behavioral evidence about attention distribution and interaction quality [29]. Jiang et al.’s UEyes dataset also demonstrates that gaze behavior varies across interface types and visual compositions [33]. These studies support the methodological value of combining subjective evaluation with gaze-based evidence. In atlas-cover design, this combined approach can help publishers and designers evaluate not only what users like, but also how users visually process cover information.

### Limitations and future prospects

First, this study focused on the immediate impact of “first-glance features” during a brief atlas-cover viewing task. Short-term aesthetic preferences may be subject to a “halo effect”; however, whether this experience diminishes due to “aesthetic fatigue” over prolonged use remains unexplored [43,44]. Longitudinal or repeated-exposure studies should therefore be conducted in future research.

Second, the findings should be interpreted with caution given the sample size and limited sample diversity. Although the final sample of 40 valid participants met the minimum requirement of the *a priori* power analysis, the participants were recruited from a single institutional context and were divided into professional and non-professional groups of 20 participants each. This sampling structure may limit the extent to which the findings can be generalized to broader populations, such as older readers, users from different educational or cultural backgrounds, and readers with varying levels of atlas-use experience. Future studies should recruit larger and more heterogeneous samples across multiple institutions and regions to further validate the robustness and external validity of the proposed design principles.

Third, the stimulus materials were reconstructed atlas covers generated from selected design categories rather than real commercial atlas covers. This approach improved experimental control by systematically manipulating color harmony, graphic style, and layout configuration while reducing potential confounds from brand identity, typography, textual semantics, and publishing context. However, it may also reduce ecological validity, because real atlas covers usually contain richer cultural symbols, commercial positioning, textual information, and market-oriented design strategies. Future research should therefore compare controlled experimental stimuli with real-world atlas covers to test whether the present findings remain stable in more naturalistic design contexts.

Fourth, this study primarily relied on oculomotor indicators, including TFD, FC, TTFF, and FFD, as behavioral measures of visual attention and information processing. These indicators should therefore be interpreted as measures of visual attention allocation and processing effort rather than as direct proxies for cognitive load. While eye-tracking is a valuable tool for analyzing visual attention and cognitive load in virtual environments [45,46], it cannot fully capture deeper cognitive or neural processing mechanisms [47]. Therefore, future research should adopt multimodal monitoring—integrating eye-tracking with physiological signals such as pupil dilation or EEG—to achieve a more comprehensive assessment of visual attention, cognitive load, and affective response [48].

Finally, the pattern of findings from brief static cover-viewing tasks may differ qualitatively in contexts involving challenging geospatial reasoning, prolonged reading, or high-pressure decision-making [49,50]. Future studies should explore the combined effects of layout configuration, color harmony, and graphic style in dynamic or interactive geospatial environments to provide more generalizable design guidelines for smart cartography [51].

## Conclusion

This study examined how color harmony, graphic style, and layout configuration influence atlas-cover evaluation by integrating perceived attractiveness ratings with eye-tracking indicators. The findings show that these visual variables shape subjective and gaze-based responses in different ways. Color harmony, graphic style, and layout configuration all significantly influenced perceived attractiveness, whereas eye-tracking indicators revealed a more selective pattern: layout configuration showed the strongest and most consistent influence on visual attention, color harmony produced smaller effects on visual exploration, and graphic style mainly affected the speed of initial visual entry. The adjusted association models showed no robust relationships between perceived attractiveness and the four AOI-averaged gaze measures, including TFD, FC, TTFF, and FFD (all $p_{Holm}\geq 0.110$). This result indicates a partial dissociation between aesthetic evaluation and gaze-based visual attention. Thus, atlas covers that are subjectively attractive are not necessarily those that guide and structure visual attention most clearly. This finding suggests that atlas-cover design should follow a dual-layer logic: layout configuration should serve as the visual-organizational scaffold for structuring visual hierarchy and guiding attention, while color harmony and graphic style should enhance affective appeal, perceptual salience, and thematic expressiveness. Overall, this study provides empirical evidence for moving atlas-cover design from intuition-based judgment toward evidence-based visual communication. The exploratory demographic analyses did not support robust moderation by professional background, age group, or map-use frequency, suggesting that these factors should be examined further in larger and more diverse samples. Future research may extend this framework to longer viewing contexts, more complex atlas designs, and dynamic or interactive geospatial media.

## Supporting information

S1 File Questionnaire and experimental instructions. This file contains the demographic questionnaire, perceived-attractiveness rating items, task instructions, and the reordered questionnaire structure corresponding to the two counterbalanced viewing rounds used in the experiment.
(XLSX)

S2 File De-identified analytical dataset. This file contains the de-identified analytical data used in the statistical analyses, including participant and cover identifiers, design-factor coding, participant-level variables, perceived-attractiveness data, eye-tracking indicators (TFD, FC, TTFF, and FFD), AOI-level visual-attention indicators, and AOI definitions. All participant information was anonymized prior to data sharing.
(XLSX)

## References

[1] Chen CS. The historical development of cartography in China. Prog Hum Geogr. 1978;2:101–20. doi: 10.1177/030913257800200106

[2] Greize L, Apele D. Book cover survey design–book sale instrument. Arts and Music in Cultural Discourse: Proceedings of the International Scientific and Practical Conference; 2017. p. 92–8.

[3] Haque E, Khan MFK, Jubair MI, Anjum J, Niloy AZ. Book cover synthesis from the summary. 2022 IEEE/ACS 19th International Conference on Computer Systems and Applications (AICCSA). IEEE; 2022. p. 1–6.

[4] Reppa I, McDougall S. Aesthetic appeal influences visual search performance. Atten Percept Psychophys. 2022;84:2483–506. doi: 10.3758/s13414-022-02567-3 PMC9630244 36241840

[5] Ooms K, De Maeyer P, Fack V, Van Assche E, Witlox F. Interpreting maps through the eyes of expert and novice users. Int J Geogr Inf Sci. 2012;26:1773–88. doi: 10.1080/13658816.2011.642801

[6] Fairbairn D, Hepburn J. Eye-tracking in map use, map user and map usability research: What are we looking for? Int J Cartogr. 2023;9:231–54. doi: 10.1080/23729333.2023.2189064

[7] Liu C, Samsudin MR, Zou Y. The impact of visual elements of packaging design on purchase intention: Brand experience as a mediator in the tea bag product category. Behav Sci (Basel). 2025;15(2):181. doi: 10.3390/bs15020181 40001812 PMC11851823

[8] Clement J. Visual influence on in-store buying decisions: An eye-track experiment on the visual influence of packaging design. J Mark Manag. 2007;23(9–10):917–28. doi: 10.1362/026725707x250395

[9] Applegate E, Carins J, Vincze L, Stainer M, Irwin C. The impact of front-of-package design features on consumers’ attention and selection likelihood of protein bars: An eye-tracking study. Food Qual Prefer. 2025;126:105427. doi: 10.1016/j.foodqual.2025.105427

[10] Guo F, Chen J, Li M, Lyu W, Zhang J. Effects of visual complexity on user search behavior and satisfaction: An eye-tracking study of mobile news apps. Univers Access Inf Soc. 2021;21(4):795–808. doi: 10.1007/s10209-021-00815-1

[11] Modi N, Singh J. Understanding online consumer behavior at e-commerce portals using eye-gaze tracking. Int J Hum–Comput Interact. 2023;39:721–42. doi: 10.1080/10447318.2022.2047318

[12] Liao H, Meng J, Zhou Y, Dong W. Investigating pupil dilation during real-world wayfinding: Effects of user, environment, and visual attention factors. Int J Digit Earth. 2025;18(1):2542960. doi: 10.1080/17538947.2025.2542960

[13] Spivey MJ, Dale R. Continuous dynamics in real-time cognition. Curr Dir Psychol Sci. 2006;15:207–11. doi: 10.1111/j.1467-8721.2006.00437.x

[14] Liao H, Wang X, Dong W, Meng L. Measuring the influence of map label density on perceived complexity: A user study using eye tracking. Cartogr Geogr Inf Sci. 2019;46:210–27. doi: 10.1080/15230406.2018.1434016

[15] Hung SM, Nieh CH, Hsieh PJ. Unconscious processing of facial attractiveness: Invisible attractive faces orient visual attention. Sci Rep. 2016;6:37117. 10.1038/srep37117 PMC5111056 27848992

[16] Clarke KC, Johnson JM, Trainor T. Contemporary American cartographic research: A review and prospective. Cartogr Geogr Inf Sci. 2019;46:196–209. doi: 10.1080/15230406.2019.1571441

[17] Yuan M. 30 years of IJGIS: The changing landscape of geographical information science and the road ahead. Int J Geogr Inf Sci. 2017;31:425–34. doi: 10.1080/13658816.2016.1236928

[18] Kuric E, Demcak P, Krajcovic M, Nguyen G. Cognitive abilities and visual complexity impact first impressions in five-second testing. Behav Inf Technol. 2024;43:3209–36. doi: 10.1080/0144929X.2023.2272747

[19] Li R, Yang Y. Consumer preference study for the interface design of traditional Chinese medicine applications using conjoint analysis method. Sci Rep. 2024;14(1):31050. doi: 10.1038/s41598-024-82279-w 39730667 PMC11680563

[20] Sweller J. Cognitive load during problem solving: Effects on learning. Cogn Sci. 1988;12:257–85.

[21] Fabrikant SI, Hespanha SR, Hegarty M. Cognitively inspired and perceptually salient graphic displays for efficient spatial inference making. Ann Assoc Am Geogr. 2010;100:13–29. doi: 10.1080/00045600903362378

[22] Barbosa AAL, de Moura JA, de Medeiros DD. Positioning of design elements on the packaging of frozen convenience food and consumers’ levels of attention: An experiment using pizza boxes. Food Qual Prefer. 2021;87:104044. doi: 10.1016/j.foodqual.2020.104044

[23] Wolfe JM, Horowitz TS. Five factors that guide attention in visual search. Nat Hum Behav. 2017;1(3):0058. doi: 10.1038/s41562-017-0058 36711068 PMC9879335

[24] Zhang N, Zhang J, Jiang S, Ge W. The effects of layout order on interface complexity: An eye-tracking study for dashboard design. Sensors (Basel). 2024;24(18):5966. doi: 10.3390/s24185966 39338711 PMC11435723

[25] Zhou C, Yuan F, Huang T, Zhang Y, Kaner J. The impact of interface design element features on task performance in older adults: Evidence from eye-tracking and EEG signals. Int J Environ Res Public Health. 2022;19(15):9251. doi: 10.3390/ijerph19159251 35954608 PMC9367723

[26] Ma C, Wang H, Wu J, Xue C. Applying gestalt similarity to improve visual perception of interface color quantity: An EEG study. Int J Ind Ergon. 2024;100:103521. doi: 10.1016/j.ergon.2023.103521

[27] Sun N, Jiang Y. Eye movements and user emotional experience: A study in interface design. Front Psychol. 2025;16:1455177. doi: 10.3389/fpsyg.2025.1455177 40177033 PMC11961869

[28] Wang W, Wen Z, Hu Z, Chen J, Gu Y, Peng Q. Evaluation method for public guidance service interface design based on improved grey H-Convex correlation model: Integrating layout aesthetics and user visual cognition. Sci Rep. 2025;15(1):15712. doi: 10.1038/s41598-025-00633-y 40325053 PMC12053586

[29] Novák JŠ, Masner J, Benda P, Šimek P, Merunka V. Eye tracking, usability, and user experience: A systematic review. Int J Hum–Comput Interact. 2024;40:4484–500. doi: 10.1080/10447318.2023.2221600

[30] Keskin M, Ooms K, Dogru AO, De Maeyer P. Exploring the cognitive load of expert and novice map users using EEG and eye tracking. ISPRS Int J Geo-Inf. 2020;9:429.

[31] Keskin M, Krassanakis V, Çöltekin A. Visual attention and recognition differences based on expertise in a map reading and memorability study. ISPRS Int J Geo-Inf. 2023;12:21.

[32] Hei Q, Yang T, Dong W, He B, Han D. Leveraging psychology and neuroscience for geospatial cognition research. Ann GIS. 2025;31(1):1–13. doi: 10.1080/19475683.2025.2451228

[33] Jiang Y, Leiva LA, Houssel PRB, Tavakoli HR, Kylmälä J, Oulasvirta A. UEyes: An eye-tracking dataset across user interface types; 2024. Dataset.

[34] Wei Y, Zheng P, Deng H, Wang X, Li X, Fu H. Design features for improving mobile health intervention user engagement: Systematic review and thematic analysis. J Med Internet Res. 2020;22(12):e21687. doi: 10.2196/21687 33295292 PMC7758171

[35] Shao J, Wu J, Tang W, Xue C. How dynamic information layout in GIS interface affects users’ search performance: Integrating visual motion cognition into map information design. Behav Inf Technol. 2022;42(11):1686–703. doi: 10.1080/0144929x.2022.2094830

[36] Thielsch MT, Scharfen J, Masoudi E, Reuter M. Visual aesthetics and performance: A first meta-analysis. Proceedings of Mensch und Computer 2019. Hamburg, Germany: ACM; 2019. p. 199–210.

[37] Li X, Li P, You H, Zhou Q, Zhao L, Huang Y, et al. Using eye tracking to explore differences and influencing factors of map reading ability in different problem situation among high school students. Comput Educ. 2025:105466.

[38] Tractinsky N, Katz AS, Ikar D. What is beautiful is usable. Interact Comput. 2000;13:127–45.

[39] Bara I, Binney RJ, Ramsey R. Investigating the role of working memory resources across aesthetic and non-aesthetic judgements. Q J Exp Psychol. 2023;76:1026–44. doi: 10.1177/17470218221101876 PMC10363947 35510887

[40] Bunch RL, Lloyd RE. The cognitive load of geographic information. Prof Geogr. 2006;58:209–20. doi: 10.1111/j.1467-9272.2006.00527.x

[41] Krassanakis V, Cybulski P. Eye tracking research in cartography: Looking into the future. ISPRS Int J Geo-Inf. 2021;10:411.

[42] Bartling M, Resch B, Reichenbacher T, Havas CR, Robinson AC, Fabrikant SI, et al. Adapting mobile map application designs to map use context: A review and call for action on potential future research themes. Cartogr Geogr Inf Sci. 2022;49:237–51. doi: 10.1080/15230406.2021.2015720

[43] Zhao J. Visual aesthetic fatigue: The impacts of beauty, novelty and type of landscape. Environ Eng Manag J. 2025;24.

[44] Bartling M, Reichenbacher T, Fabrikant SI. Leveraging map use context for advancing cartography in the 21st century. Proceedings of the ICA. vol. 5. Göttingen, Germany: Copernicus Publications; 2023. 2 p.

[45] Khan R, Vernooij J, Salvatori D, Hierck BP. Assessing cognitive load using EEG and eye-tracking in 3-D learning environments: A systematic review. Multimodal Technol Interact. 2025;9(9):99. doi: 10.3390/mti9090099

[46] Shadiev R, Li D. A review study on eye-tracking technology usage in immersive virtual reality learning environments. Comput Educ. 2023;196:104681.

[47] He B, Zhang H, Qin T, Shi B, Wang Q, Dong W. A simultaneous EEG and eye-tracking dataset for remote sensing object detection. Sci Data. 2025;12(1):651. doi: 10.1038/s41597-025-04995-w 40246854 PMC12006373

[48] Qin T, Fias W, Van De Weghe N, Huang H. Recognition of map activities using eye tracking and EEG data. Int J Geogr Inf Sci. 2024;38:550–76. doi: 10.1080/13658816.2024.2309188

[49] Zhang M, Gong Y, Deng R, Zhang S. The effect of color coding and layout coding on users’ visual search on mobile map navigation icons. Front Psychol. 2022;13:1040533. doi: 10.3389/fpsyg.2022.1040533 36582311 PMC9792669

[50] Yang B, Li H. A visual attention model based on eye tracking in 3D scene maps. ISPRS Int J Geo-Inf. 2021;10(10):664. doi: 10.3390/ijgi10100664

[51] Habib M, Okayli M. An overview of modern cartographic trends aligned with the ICA’s perspective. Rev Int Geomat. 2023;32:1–16.

